# Cell-Free DNA as a Biomarker in Autoimmune Rheumatic Diseases

**DOI:** 10.3389/fimmu.2019.00502

**Published:** 2019-03-19

**Authors:** Bhargavi Duvvuri, Christian Lood

**Affiliations:** Division of Rheumatology, Department of Medicine, University of Washington, Seattle, WA, United States

**Keywords:** cell-free DNA, biomarker, autoimmune rheumatic diseases, systemic lupus erythematosus, rheumatoid arthritis

## Abstract

Endogenous DNA is primarily found intracellularly in nuclei and mitochondria. However, extracellular, cell-free (cf) DNA, has been observed in several pathological conditions, including autoimmune diseases, prompting the interest of developing cfDNA as a potential biomarker. There is an upsurge in studies considering cfDNA to stratify patients, monitor the treatment response and predict disease progression, thus evaluating the prognostic potential of cfDNA for autoimmune diseases. Since the discovery of elevated cfDNA levels in lupus patients in the 1960s, cfDNA research in autoimmune diseases has mainly focused on the overall quantification of cfDNA and the association with disease activity. However, with recent technological advancements, including genomic and methylomic sequencing, qualitative changes in cfDNA are being explored in autoimmune diseases, similar to the ones used in molecular profiling of cfDNA in cancer patients. Further, the intracellular origin, e.g., if derived from mitochondrial or nuclear source, as well as the complexing with carrier molecules, including LL-37 and HMGB1, has emerged as important factors to consider when analyzing the quality and inflammatory potential of cfDNA. The clinical relevance of cfDNA in autoimmune rheumatic diseases is strengthened by mechanistic insights into the biological processes that result in an enhanced release of DNA into the circulation during autoimmune and inflammatory conditions. Prior work have established an important role of accelerated apoptosis and impaired clearance in leakage of nucleic acids into the extracellular environment. Findings from more recent studies, including our own investigations, have demonstrated that NETosis, a neutrophil cell death process, can result in a selective extrusion of inflammatory mitochondrial DNA; a process which is enhanced in patients with lupus and rheumatoid arthritis. In this review, we will summarize the evolution of cfDNA, both nuclear and mitochondrial DNA, as biomarkers for autoimmune rheumatic diseases and discuss limitations, challenges and implications to establish cfDNA as a biomarker for clinical use. This review will also highlight recent advancements in mechanistic studies demonstrating mitochondrial DNA as a central component of cfDNA in autoimmune rheumatic diseases.

## Introduction

In 1948, Mandel and Metais were the first to report on the presence of cfDNA in human plasma (1). However, it was not until the discovery of high levels of cfDNA in systemic lupus erythematosus (SLE) patients in 1966 (2), that the interest in cfDNA as a potential biomarker for autoimmune diseases started. Early reports were flawed by leukocyte release of DNA during coagulation, with a major breakthrough in the field upon recognizing plasma as a better source of pathological cfDNA, with undetectable levels in healthy volunteers (3–5). These early observations were followed by the demonstration of cfDNA in other autoimmune diseases including rheumatoid arthritis (RA) (6). However, with the advent of more sensitive methods, cfDNA was also detected in plasma of healthy individuals, albeit at very low levels. This observation, together with the temporal association of the increased levels of cfDNA with disease activity in patients with SLE and RA, led to the proposition that cfDNA can be a potential biomarker for autoimmune diseases. Subsequent, technological advancements such as fluorometric assays and real-time PCR led to the simple and rapid quantification of cfDNA, also providing information on the intracellular origin of cfDNA, such as the mitochondria. Despite the initial excitement and technological advancements in cfDNA quantification, there was not a substantial interest in cfDNA as a biomarker for autoimmune diseases, until more recently with the increasing understanding into the role of DNA-sensing receptors in inflammation and autoimmunity, especially in SLE and RA. In this review, we will give an overview of basic biology of cfDNA, followed by evolution of cfDNA in SLE and RA as a biomarker of diagnosis, disease activity and progression, and as a prognostic marker of treatment response.

## Mechanisms of cfDNA Release

Though our understanding of mechanisms contributing to the generation of cfDNA is evolving with several novel pathways described in recent years (7), it is still unclear which, if any, of the current models account for the elevated levels of cfDNA observed in patients with rheumatic disease. Beneath we will highlight key cell death processes and active release mechanisms, and their potential implication in rheumatic disease.

## Apoptosis

Apoptosis, also known as programmed cell death, is an essential part of physiological maintenance of cellular homeostasis that eliminates unwanted and damaged cells. Apoptosis is executed by effector caspases that are activated in extrinsic and intrinsic-pathways triggered by death-receptors and intracellular stimuli such as oxidative stress and DNA damage, respectively. Activation of caspases, a mark of an irreversible commitment to apoptosis, results in a proteolytic cascade leading to several characteristic morphological and biochemical changes in apoptotic cells that include cell and nuclear shrinkage, DNA fragmentation and lipid re-distribution (8, 9). Taking cues from these alterations as find-me signals, especially upon the exposure of phosphatidylserine onto the cell surface, apoptotic debris under normal conditions, is promptly recognized and cleared by phagocytes in a non-inflammatory process called efferocytosis (10). The clearance of apoptotic cells exerts powerful anti-inflammatory and immunosuppressive effects (11). In contrast, impaired clearance of apoptotic material and/or increased cell death process lead to an accumulation of intracellular antigens and DNA extracellularly, which long-term can lead to autoinflammatory responses (12–14). Inter-nucleosomal fragmentation of DNA into double-stranded DNA fragments of 180–200 bp by calcium-dependent endonucleases is the biochemical hallmark of apoptosis. The fragmented DNA is detectable as a ladder pattern when subjected to gel electrophoresis (15). Multiple characteristics of cfDNA suggest that it is reminiscent of apoptotic DNA. cfDNA, like apoptotic DNA, is a highly fragmented, low molecular weight double stranded DNA with an average size of ~150–200 bp in length, a size corresponding to nucleosomal DNA, and exhibits a ladder pattern on gel electrophoresis as multiples of nucleosomal units (16). SLE, a disease characterized by increased apoptosis and impaired clearance of apoptotic cells (17, 18), shows evidence of low molecular weight cfDNA with an apoptosis-like size distribution pattern. DNA purified from SLE plasma formed discrete bands, predominantly with a unit size of ~200 bp, characteristic of DNA found in oligonucleosomes (19). In another study that isolated DNA from the DNA-anti-DNA antibody immune complexes in sera of SLE patients, a strong correlation was observed between low molecular weight DNA sizes (both 30–50 and 150–200 bp), disease activity, and the frequency of renal disease (20). Genome-wide sequencing identified that plasma DNA in SLE patients exhibit size shortening (≤115 bp in length) that correlated with SLEDAI and anti-double stranded DNA (dsDNA) antibody level. In addition, IgG-bound DNA fragments of SLE patients are shorter (≤115 bp) than non-IgG bound DNA (19).

## Necrosis

The presence of also high molecular weight cfDNA led to the proposition that necrosis could be the release mechanism (21, 22). Necrosis is an accidental form of cell death in response to physical or chemical injury characterized by cell swelling and rapid loss of plasma membrane integrity, leading to the release of intracellular contents. Necrosis results in non-specific digestion of chromatin, thus enabling release of high molecular weight DNA of many kilo base pairs (23). Necrotic release of cfDNA could be relevant in conditions including trauma, injury and sepsis, where levels of cfDNA were associated with the severity of trauma and post-traumatic complications (24, 25), injury (26, 27), and mortality in patients with sepsis (28, 29). Although the role of necrosis in the elevated levels of cfDNA observed in patients with rheumatic disease has not been carefully investigated, studies in SLE patients suggest that necrotic cell death can be a major source of cfDNA. Intracellular ATP concentration is one of the factors that determines the cell's fate to undergo cell death via apoptosis or necrosis. Interestingly, CD4+ T cells from SLE patients are characterized by ATP depletion due to persistent mitochondrial hyperpolarization, which subsequently results in the uncoupling of oxidative phosphorylation i.e., continued production of reactive oxygen intermediates in the absence of ATP synthesis. This ATP depletion results in a diminished activation-induced apoptosis and sensitization of CD4+ T cells to undergo necrosis, thus enabling the release of cellular contents, including cfDNA, into the extracellular space (30, 31).

## NETosis

In response to microbes, as well as sterile inflammation, neutrophils can undergo a unique form of programmed cell death known as NETosis. It results in the extrusion of a web-like structure of nuclear-derived decondensed DNA coated with histones, granular proteins, and cytoplasmic proteins into the extracellular space. Since the extracellular chromatin fibrils could entangle microbes, the structures were named neutrophil extracellular traps (NETs) (32). Unlike suicidal NETosis described above, which results in the death of neutrophils, neutrophils may undergo vital NETosis, a process in which they only extrude a small amount of DNA, preferentially mitochondrial DNA (mtDNA), allowing for the neutrophil to remain alive and continue to exert antimicrobial actions (32, 33). Following the discovery of NETosis, and extrusion of DNA by neutrophils, several other cell types have been identified to release extracellular traps (34–37), a process termed ETosis. Considering dsDNA being the structural backbone of NETs, in the conditions of excessive NETosis (38) or impaired clearance (39, 40), remnants of NETs could account for elevated levels of circulating cfDNA. Consistent with this proposition, NET deposition was found to be associated with levels of cfDNA in various pathological conditions including SLE, rheumatoid arthritis, cancer, and transfusion-related acute lung injury (38, 41–44).

## Pyroptosis

Pyroptosis is a lytic form of inflammatory cell death induced by inflammasome activation in response to diverse pathogen and host-derived danger signals (45). Inflammasome activation leads to the processing and activation of inflammatory caspases, which in turn mediate the downstream inflammatory processes that include the processing of pro-inflammatory cytokines, IL-1β and IL-18, and lytic events associated with pyroptosis (46–48). Early during this cell death, pores are formed in the cell membrane in a caspase-dependent manner ultimately resulting in cell lysis and the release of intracellular inflammatory contents, including IL-1β and IL-18 (49). An unidentified-caspase1-activated nuclease leads to DNA fragmentation during pyroptosis (49). Hence, in conditions associated with caspase-mediated induction of IL-1β and IL-18, the disruption of cell membrane can contribute to release of cfDNA. Tan et al. (50) demonstrated that HIV patients who develop tuberculosis-associated immune reconstitution inflammatory syndrome following cART therapy exhibit increased inflammasome activation, represented by the increased plasma levels of IL-18 that correlate significantly with plasma levels of cell-free mtDNA (cf-mtDNA), a finding suggestive of pyroptosis in cfDNA release (51).

## Active Secretion

Other than cell death, cells could actively secrete DNA in the form of extracellular vehicles (EVs), including exosomes and microparticles (microparticles). This cfDNA, present in membrane-bound EVs, may be protected from degradation by nucleases and can be released through the breakdown of EVs. Exosomes are small 30–100 nm vesicles released from the fusion of multivesicular bodies of endosomal origin with plasma membranes. The composition of exosomes includes nucleic acids, proteins, lipids and other metabolites (20). Through their biologically active components, including DNA, exosomes were shown to modulate various physiological and pathological processes (52, 53). A recent study by Fernando et al. (54), demonstrated that more than 93% of amplifiable plasma cfDNA is present in exosomes and the size of the majority of exosomal DNA is <200 bp, consistent with the size of cfDNA reported in plasma from patients with rheumatic disease. Alternatively, a study by Kahlert et al. (55) demonstrated that exosomes could also be the source of high molecular weight DNA (>10 kb). Interestingly, they could not detect any PCR amplifiable products in serum depleted of exosomes; also suggesting that the majority of cfDNA is present in exosomes. These findings suggest that the content of exosomes could vary depending on their cellular origin and the stimuli modulating their release from cells. Microparticles (MPs) or shedding vesicles, a small membrane-bound 100–1,000 nm vesicles can be released from apoptotic cells as blebs or can be actively secreted from living cells. DNA from MPs shows laddering pattern, resembling nucleolytic cleavage of apoptotic cells (56). In general, SLE patients have a higher frequency of pro-inflammatory MPs in the circulation (57, 58). MPs, in particular if released from activated platelets, could also be the source of cf-mtDNA (57, 59, 60). Importantly, upon platelet activation, mitochondria (either naked or localized within MPs) are extruded together with the bactericidal enzyme phospholipase A2, enabling digestion of membranes, allowing the pro-inflammatory mtDNA to escape unto the extracellular space (59). Indeed, levels of mtDNA increase concomitantly with levels of phospholipase A2 in platelet storage bags, with mtDNA levels being associated with adverse transfusion reactions (59).

## Clearance of cfDNA

We have so far only considered the extrusion of cfDNA. Another important component in generating elevated levels of circulating cfDNA is impaired clearance mechanisms. A rapid clearance of cfDNA is critical to prevent not only inflammation, but also the potential development of autoimmunity toward DNA, as seen in SLE. Early studies on cfDNA clearance revealed that under physiological conditions, cfDNA is rapidly degraded by endonucleases and eliminated from the circulation through several organ systems, including the spleen, liver, and kidney (51, 61–63). Many factors can influence the ability of DNases to clear cfDNA, including whether the cfDNA is complexed with proteins, nucleosomes and/or antibodies, as well as whether the cfDNA is in free circulating form or encapsulated within membrane-enclosed particles, including exosomes, MPs and apoptotic bodies. Further, based on its intracellular origin, e.g., either nuclear or mitochondrial, cfDNA can exhibit different structural characteristics and stability (64–66).

Efficient degradation of free and protein-bound DNA i.e., nucleosomal DNA in plasma/serum is carried out by extracellular nuclease homologs, DNase I and DNase I-like III (DNase I L3), respectively (67, 68). While, DNase I efficiently cleaves free DNA, the digestion of nucleosomal DNA present in extracellular space and/or sequestered in MPs is majorly performed by DNase I L3 (69, 70). These specific activities of DNase I L3 are attributed to the presence of short, positively charged peptide in the carboxyl-terminal of DNase I L3 (71). Given the importance of DNase I and DNase I L3 in the degradation of circulating DNA, several studies have investigated the role of these nucleases in the context of SLE, a disease characterized by reduced ability to clear cellular debris. Abnormalities of DNase I activity reported in lupus include low serum DNase I activity particularly in patients with renal disease (72, 73), increased serum levels of DNase I inhibitors like G-actin that associated with the high titers of antinuclear antibodies (74), and novel mutations in the enzyme accompanied by elevated titers of anti-dsDNA antibodies (75, 76). Autoantibodies, including anti-dsDNA antibodies, can protect DNA from DNase I digestion (40, 77). Further, anti-DNase antibodies as observed in SLE, were shown to interfere with the enzyme activity, leading to low serum DNase activity (78). As briefly mentioned above, molecules interacting with the DNA may also affect the ability of the DNA to be recognized and degraded by DNase I. Cationic proteins like cathelicidin LL37, human neutrophil peptides and IL-26, protect cfDNA by forming insoluble aggregates through their charge interactions (79, 80) and mitochondrial transcription factor A (TFAM), a mitochondrial packaging protein is involved in the protection of mtDNA from nuclease degradation (81). In addition, defects in the cofactors that promote the DNase I activity (82–85) can also cause or perpetuate the decreased DNase I activity. For instance, complement component C1q, a deficiency of which is strongly associated with the genetic susceptibility of SLE, was shown to promote DNase I activity in degrading necrotic cell-derived chromatin (82). These cofactors likely promote the DNase I activity either by displacing DNA binding proteins from chromatin thus allowing the access to cleavage sites on DNA or by stabilizing DNase I on the target. With regards to DNase I L3, a homozygous loss-of-function variant mutation in *DNASE1L3* gene, identified in several families of pediatric-onset SLE patients, was found to be associated with a higher frequency of anti-dsDNA antibodies and lupus nephritis (86). Another study reported two unique *DNASE1L3* gene mutations in families with autosomal-recessive hypocomplementemic urticarial vasculitis syndrome (HUVS) (87). Incidentally, HUVS is more often associated with SLE, with >50% of HUVS patients often developing SLE (87). In this particular study, 3 of 5 children with HUVS carrying a homozygous frame-shift mutation in *DNASE1L3* gene developed severe symptoms of SLE accompanied by anti-dsDNA antibodies (87). In addition to extracellular nucleases, TREX1, a major mammalian intracellular DNase with a preference for single-stranded DNA (ssDNA) substrates, can be involved in the degradation of cfDNA that translocate to the cytosol through carrier proteins. TREX1 is defective in the degradation of oxidized substrates such as oxidized mtDNA, which are preferentially from SLE neutrophils (38, 88). Hence, in conditions like lupus, the persistent presence of oxidized cf-mtDNA in the cytosol of immune cells can potentially activate inflammatory pathways. TREX1 variant mutations are also reported in SLE (89, 90). Finally, complement C1q, as well as other complement components also play an important role in opsonizing dead cells or extracellular debris for phagocytosis, thus efficiently removing cfDNA from the circulation (82, 91). Other opsonins, including serum amyloid *P* component (92), IgM (93, 94), C-reactive protein (CRP) (95, 96), and Mannan Binding Lectin (97) serve similar functions in clearance of dying cells, with deficiencies in either one of the opsonins commonly leading to accumulation of cfDNA (98).

## Inflammatory Potential of cfDNA

Under physiological conditions, cfDNA is normally not inflammatory due to its rapid degradation as well as its inability to access intracellular DNA sensors. Consistent with this proposition, cfDNA failed to induce immune responses from plasmacytoid dendritic cells (pDCs), which are otherwise potent responders to microbial nucleic acids (79, 99, 100). Initially, this tolerance to self-DNA was thought to be due to the sequence composition differences between self- and microbial DNA. However, numerous studies have shown that self-DNA can be immunostimulatory provided it has access to intracellular DNA sensors (101–103). These carrier proteins, often elevated in inflammatory conditions (79, 104), can facilitate the uptake of DNA and also protect the DNA from degradation, thus promoting the induction of pro-inflammatory responses.

## Based on Complexation With Carrier Proteins

In SLE, anti-dsDNA autoantibodies are one of the prominent carrier molecules of cfDNA into cells. Among others, anti-dsDNA antibodies, through their interaction with Fcγ receptor II (FcγRII) facilitate the receptor-mediated endocytosis of DNA into the TLR9-containing endosomal compartments of pDCs, eliciting a robust induction of interferon (IFN)-α (IFN-α), a cytokine markedly elevated in SLE and associated with disease activity (105). In an attempt to understand the role of the anti-dsDNA antibodies in promoting DNA-immune complex (IC)-mediated inflammation, Means et al. (104) undertook a series of experiment to dissect the role of the autoantibodies. Whereas, neutralization of FcgRII abrogated the immune reactivity of ICs, this could be rescued through a liposomal carrier. In all, these elegant experiments suggested that the primary role of IgG autoantibodies is to facilitate the entry of DNA into cells and are not obligatory for the immunogenicity of DNA (104). Later, a study by Lande et al. (79), provided evidence that cationic microbial peptides that are released in the context of NETosis, confer the immunogenicity to DNA-ICs by protecting DNA from nuclease degradation and facilitating uptake. Further, their data suggest that complexes of self-DNA-antimicrobial peptides (i.e., LL37) constitute the immunogenic core of the DNA-ICs in SLE, since anti-DNA antibodies alone were not sufficient to confer immunogenicity to DNA. LL37 is highly expressed in the circulation of SLE patients (106). LL37 stably binds to DNA through charge interactions between the unique cationic α-helical residues of LL37 and anionic phosphate backbone of DNA, thus forming insoluble DNA aggregates that are resistant to nuclease degradation (107). Antimicrobial peptides, including human neutrophil peptides, seem to function synergistically with LL37 to promote pDC activation by DNA-ICs (79). IL-26, a cationic amphipathic cytokine secreted by Th17 cells seems to stabilize and thereby promote the immunogenicity of extracellular DNA (80). IL-26, through its clusters of cationic charges, binds, and aggregates human DNA, thus forming insoluble particles that are resistant to extracellular degradation. Further, Meller et al. (80) showed that IL-26-DNA complexes could induce IFN-α secretions from pDCs in TLR9-dependent manner, and the internalization of complexes is mediated by the attachment of IL-26 cationic residues to heparan-sulfate proteoglycan on the cell membrane. Later, Poli et al. (108) showed that IL-26 shuttles different forms of extracellular DNA (genomic DNA, mitochondrial DNA, and NETs) into the cytosol of monocytes and promotes cyclic GMP-AMP synthase (cGAS)-STING- and inflammasome-dependent pro-inflammatory responses. Further, high levels of IL-26-DNA complexes have been found in patients with anti-neutrophil cytoplasmic antibody-associated vasculitis (108), RA (109), psoriasis (110), and Crohn's disease (111). cfDNA can also be translocated into cell when bound to DNA-binding high mobility group box proteins, like high mobility group 1 (HMGB1) and TFAM that are released as DAMPs into extracellular space from damaged and dying cells and also during inflammation (112, 113). These molecules can specifically engage receptor for advanced glycation end products (RAGE) on pDCs to elicit TLR9-dependent IFN-α secretions for DNA ligands (114, 115). Further, DNA bound by these molecules can presumably undergo conformational changes that allow them to bind and activate a cytosolic DNA sensor, cGAS to initiate STING-mediated type-I IFNs (116).

## Based on Oxidative Status of cfDNA

Another important aspect that can render cfDNA inflammatory is the presence of oxidized nucleotides, as DNA oxidation, whether bound to cationic peptides or not, is recognized as a danger associated molecular pattern. Further, the immunogenicity of DNA seems to depend on the degree of oxidation (117). Elevated levels of oxidized cfDNA is observed in various inflammatory conditions (118), supporting the role of oxidative status in promoting the inflammatory potential of DNA. DNA can be oxidized by free oxygen radicals or reactive oxygen species (ROS) that are generated during cell death processes (119), thus resulting in the release of oxidized cfDNA. We have shown that neutrophil cell death i.e., NETosis in response to ribonucleoprotein-containing ICs (RNP ICs), is dependent on mitochondrial ROS and the released NETs are enriched in oxidized interferogenic mtDNA (38). Alternatively, cfDNA can be oxidized by free oxygen radicals or ROS that are released by activated phagocytic cells at sites of inflammation (120). Oxidized DNA undergo structural changes due to the base modifications introduced by free radicals. As a result, the oxidized DNA is more resistant to nuclease degradation, as compared to unmodified DNA, thus making the oxidized DNA available to initiate pro-inflammatory responses (117). Although ROS can cause diverse arrays of DNA base modifications, a lesion to guanine residue identified as 8-hydroxy-2′-deoxyguanosine (8-OHdG) remains the most abundant and well-characterized DNA lesion (121). In fact, 8-OHdG was identified as a marker to determine the oxidative stress in various pathological conditions (122). Oxidized DNA was shown to activate various inflammatory pathways including, cGAS-STING, TLR9, and NLRP3 inflammasome pathways (38, 117, 123, 124). Further, oxidation by ROS increases the antigenicity of DNA, as demonstrated by the enhanced reactivity of SLE sera with oxidized DNA compared to native DNA (88, 125, 126). Consistent with the above observation, DNA contained within SLE immune complexes show an accumulation of 8-OHdG, indicating that oxygen radicals play a key role in the SLE pathology by modulating the antigenic nature of DNA in circulation (125, 126).

## Based on Intracellular Origin

The intracellular origin of cfDNA, e.g., either from nucleus or mitochondria, can also influence the inflammatory potential of cfDNA. MtDNA, unlike nuclear DNA, is a potent inflammatory trigger (26, 38, 123, 127–130). MtDNA, due to its prokaryotic origin, holds many features that are similar to bacterial DNA, including the presence of a relatively high content of unmethylated CpG motifs, which are rarely observed in nuclear DNA (131). The unmethylated CpG motifs are of particular importance as TLR9, the only endolysosomal DNA-sensing receptor, has a unique specificity for unmethylated CpG DNA. As such, mtDNA was shown to activate neutrophils through TLR9 engagement (26, 103). Thus, unless coupled to carrier proteins, mtDNA, but not nuclear DNA, can be recognized as a danger-associated molecular pattern inducing pro-inflammation through TLR9. Collins et al. (129), reported that intra-articular injection of mtDNA induces arthritis *in vivo*, proposing a direct role of mtDNA extrusion in the disease pathogenesis of RA. Also, the oxidative status of mtDNA makes it highly inflammatory (123, 127). MtDNA, in contrast to nuclear DNA, is characterized by elevated basal levels of 8-OHdG, a marker of oxidative damage (66). The high content of oxidative damage in mtDNA is primarily attributed to the close proximity of mtDNA to ROS and relatively inefficient DNA repair mechanisms that can lead to the accumulation of DNA lesions (132, 133). We, as well as others, have shown that oxidative burst during NETosis can oxidize mtDNA and the released oxidized mtDNA by itself, or in complex with TFAM, can generate prominent induction of type I IFNs (38, 88, 117). Oxidative status of mtDNA renders its resistant to degradation by DNases such as TREX1, enabling it to activate multiple pro-inflammatory pathways (95). Oxidized mtDNA generated during programmed cell death is not limited to activate TLR9, but was shown to also engage the NRLP3 inflammasome, leading to the production of pro-inflammatory cytokines, IL-1β, and IL-18 (123, 127, 128, 130). MtDNA can also be recognized by cyclic GMP-AMP synthase (cGAS), a cytosolic dsDNA sensor to initiate a STING-IRF3-dependent pathway that in turn orchestrates the production of type I IFNs (134).

## DNase I as a Therapeutic Agent

Considering the role of DNase I in promoting the clearance of autoantigenic DNA and perhaps even the destruction of DNA in ICs, the therapeutic potential of DNase I has been explored. Initial studies by Johnson and colleagues in the 1950s demonstrated that bovine pancreatic DNase I is harmless to humans and is a very poor antigen (135). It was used as a liquefaction agent to treat disease conditions associated with exudative responses to inflammation and infection (136). These studies by Johnson et al., (135, 136) laid basis for the first therapeutic application of bovine DNase I to treat SLE where patients injected with DNase I showed improvement in clinical symptoms, including a rapid fall in the ESR and also in the levels of autoantibodies specific to DNA-containing antigens but not in other autoantibodies. However, contrary to the initial reports, bovine DNase I was found to be antigenic and patients developed antibodies toward it 4–6 weeks following the administration, thus precluding the therapeutic usage of DNase I (137). Meanwhile, animal studies with recombinant mouse DNase I to treat SLE in NZB/W F1 hybrid mice prompted further interest in DNase as therapeutic agent for SLE (138). Mice treated with DNase I from the early phases of disease development (4 months of age) had a prolonged survival of about a month that was paralleled by the delay in the selective formation of anti-DNA antibodies but not in other autoantibodies or total IgG levels. Further, a concomitant rise in anti-DNA antibodies was observed in urine suggesting the destruction of DNA-containing ICs in kidney by DNase treatment. The DNase-treated mice developed less severe glomerulonephritis compared to control mice. Anti-DNase antibodies were formed in all DNase-treated mice, although they did not rise and remained low throughout the treatment period. The effect of these anti-DNase antibodies on the DNase function was not clear. In addition, it was not clear why sustained effect of DNase could not be seen and if more enzyme could have been beneficial given the rise in DNase inhibitors with inflammation. Nevertheless, much more dramatic changes in clinical course were observed in a group of mice treated with DNase for 3 weeks at the peak of their disease activity (7 months of age). DNase-treated mice displayed significantly lower levels of serum creatinine and less proteinuria compared to controls accompanied by remarkably less severe histopathological changes in the kidney, suggesting that DNA-containing ICs might still be involved in the advanced stage of disease course. Since animals were killed at the end of experiment, the long-term effect of DNase treatment is not clear. The study also suggests that destruction of established ICs might be an effective therapeutic option for SLE, thus preventing the stimulation of autoreactive B cells. Investigations into the human usage of DNase I in SLE were prompted by the discovery of recombinant human DNase I (rhDNase) as a potential agent to attenuate the sputum viscosity in cystic fibrosis patients (139). RhDNase administered by an inhalation method was found to be safe and well-tolerated in patients with cystic fibrosis (140–142). Subsequently, a 40-day, phase 1b placebo-controlled clinical trial conducted in SLE patients with lupus nephritis showed that rhDNase I is safe in SLE patients, and that the treatment was not associated with the development of anti-rhDNase antibodies. Patients were given an initial single dose (25 or 125 μg/kg) of intravenous injection followed by 10 subcutaneous injections of rhDNAse I. However, the treatment did not result in a significant improvement in markers of disease activity including the levels of serum dsDNA antibodies, levels of complement components C3 and C4, levels of circulating immune complexes, serum cytokines levels (IL-6, IL-10, and TNF-α) and there was no change in the immune complex deposition in skin biopsies pre- and post-treatment. This lack of clinical efficacy could partly be explained by the observation that rhDNase at bioactive serum concentrations was maintained for only a few hours at both doses, and thus future studies should investigate the effect of dose or regimen that allows the maintenance of rhDNase at concentrations capable of serum hydrolytic activity for prolonged time periods. The absence of neutralizing antibodies to rhDNase unlike to bovine DNase I suggests the potential possibility for long-term therapy (143).

## Cell-Free DNA in Rheumatic Diseases

### Systemic Lupus Erythematosus

SLE is a prototype autoimmune disease that effects multiple tissues and organs systems, including skin, joints, and kidney. Though known to have both environmental and genetic components, the etiology of the disease is not fully understood. Among several markers implicated in the SLE pathogenesis, the role of dsDNA is especially interesting. In the majority of the SLE literature, the pathological role of dsDNA in the disease pathogenesis is discussed from the perspective of dsDNA-containing immune complexes, frequently found in SLE patients, partaking in the development of lupus nephritis. The prevailing idea is that these immune complexes activate complement factors and Fc gamma receptor-bearing cells to initiate pathological inflammatory responses. In fact, anti-dsDNA antibodies are listed among the classification criteria for diagnosing SLE in accordance with the American College of Rheumatology, and the Systemic Lupus International Collaborating Clinics classification criteria (144, 145). Other than the diagnostic utility, increasing research into the potential role of DNA sensing receptors (146) to initiate multiple pro-inflammatory pathways resulting in the secretion of SLE-associated type I interferons, brought dsDNA back into the center stage of the SLE pathogenesis. Subsequently, the interest to detect, quantify and/or characterize cfDNA in plasma/sera of SLE patients emerged. In the section below, we will present a glimpse of how the research into the field of cfDNA has evolved in SLE, with initial studies mainly focusing on the detection and quantification of cfDNA followed by studies to associate cfDNA levels to disease activity, progression and/or for monitoring treatment response. Finally, we will touch upon the application of advanced sequencing techniques to determine characteristics of plasma DNA, all with a common goal to explore the diagnostic and prognostic potential of cfDNA for SLE. Major findings of cfDNA in SLE as discussed in the following sections are summarized and listed in Table 1.

**Table 1:** Cell-free DNA research in Systemic lupus erythematosus and Rheumatoid arthritis.

**References**	**Year of publication**	**Patients (*n*)**	**Healthy controls, HC (*n*)**	**Method**	**cfDNA source**	**Observation**
Tan and Kunkel (2)	1966	SLE (95)	30	Gel diffusion precipitin test	Serum	Frequency of positive test for cfDNA: SLE 11.5%, liver disease 15%, lymphosarcoma 3%, and none were observed in HC
		Leukemia, lymphosarcoma, and lymphoma (29)				cfDNA positivity fluctuate with disease activity
		Multiple myeloma (15)				
		Acute rheumatic fever (9)				
		RA (17)				
		Liver disease (40)				
		Miscellaneous (myocardial infarction, renal disease, lung disease, infection, carcinoma) (72)				
Barnett (3)	1968	Serum tested	14	Quantitative complement-fixation test	SF and Serum	↑ cfDNA in the sera of patients with SLE, RA, systemic mastocytosis, uremia with chronic glomerulonephritis compared to HC
		RA (6)				↑ cfDNA in SFs of patients with SLE, RA, Gout
		SLE+Nephritis (6)				
		SLE-Nephritis (7)				
		Systemic Mastocytosis (1)				
		Uremia with Chronic Glomerulonephritis (1)				
		Vasculitis, Local (1)				
		Rheumatic Heart (1)				
		Psoriasis+RA (2)				
		Gout (1)				
		Scleroderma (1)				
		SF tested				
		SLE (1)				
		RA (3)				
		Gout (2)				
		Reiter's (1)				
		Infection (1)				
Koffler et al. (4)	1973	SLE (60)	56	Hemagglutination inhibition test	Serum	↑ cfDNA in sera of patients with SLE and RA compared to HC and other diseases
		RA (54)				Association with disease severity
		Chronic glomerulonephritis (40)				
		Leukemia (19)				
		Malignant tumors (20)				
		Hospital diseases (99)				
Davis and Davis (147)	1973	SLE (44)	83	Counterimmuno-electrophoresis	Plasma	Frequency of positive test for cfDNA: 1.2% in HC, 3.2% in RA, 4.2% in SLE, 1.4% preoperative, 44.0% post-operative
		RA (28)				
		Newborn cord bloods (36)				
		Surgery				
		Preoperative (71)				
		Post-operative (50)				
		Nonsurgical (278)				
		Miscellaneous (60)				
Leon et al. (148)	1977	RA (70)	61	Radioimmunoassay	Serum	↑ cfDNA in RA patients compared to HC
						↑ cfDNA levels in RA patients with active disease for <10 years, seronegative for rheumatoid factor
Steinman (149)	1979	SLE (43)	None	Modified counterimmuno-electrophoresis	Plasma	Frequency of positive test for cfDNA: 80% in SLE with CNS involvement and vasculitis, 20% in SLE with dermal vasculitis
		SLE+CNS involvement (12)				5.5% in active SLE, none in other rheumatological disorders and SLE patients on treatment
		SLE+Systemic Vasculitis (8)				
		SLE+Dermal Vasculitis (5)				
		Other rheumatological disorders and SLE on treatment (53)				
Raptis and Menard (150)	1980	inactive SLE (5)	3	Nick translation on purified cfDNA	Plasma	↑ cfDNA levels in active SLE patients compared to steroid-inactive SLE and HC
		active SLE (2)				cfDNA positivity fluctuate with disease activity
		RA (2)				
		DM (1)				
Leon et al. (151)	1981	Seropositive RA (26)	95	Radioimmunoassay	Serum and SF	↑ cfDNA in SF and serum of RA, gout and pseudogout
		Seronegative RA and variants (21)				Temporal correlation between serum and SF cfDNA during active disease in RA patients
		Non-classified and mono- and oligoarticular RA (6)				
		Gout and pseudogout (6)				
		OA (29)				
		OA+chonrocalcinosis (4)				
		Post-traumatic arthropathy (14)				
Klemp et al. (152)	1981	Clinically active SLE (43) with specific organ or system involved	58	PAGE and fluorimetric scan on purified cfDNA	Plasma	Frequency of subjects with cfDNA <10 ng/ml: Clinically active SLE: 88%; Clinically inactive SLE: 82%; HC: 81%
		Skin (8)				Frequency of subjects with cfDNA >10 ng/ml: Clinically active SLE: 11.6%; Clinically inactive SLE: 17%
		Musculoskeletal system (20)				
		Kidney (5)				
		Nervous system (3)				
		Cardiovascular system (1)				
		Nonspecific (constitutional symptoms) (6)				
		Clinically inactive (53)				
Morimoto et al. (153)	1982	SLE (28)	5	^32^P-phosphate incorporation into 5′ ends of DNA	Serum	↑ concentrations of DNA in DNA/anti-DNA immune complexes of SLE patients
		RA (4)				DNA in DNA/anti-DNA immune complexes of SLE patients correlate with disease activity
McCoubrey-Hoyer et al. (154)	1984	SLE+Nephritis at the time of blood sampling (10)	20	Counterimmuno-electrophoresis on purified cfDNA	Plasma	↑ concentrations of cfDNA in SLE patients compared to HC
		SLE+Nephritis in the past that was inactive at the time of blood sampling (9)				cfDNA levels in the plasma of SLE patients did not correlate with nephritis.
		SLE-Nephritis (12)				
Hajizadeh et al. (155)	2003	RA (54)	30 and 22	PCR and SDS–polyacrylamide-gel electrophoresis	SF and Plasma	PCR-amplifiable mtDNA fragments detected in SF of RA patients but not in HCs
						↑ of cfDNA in plasma of RA patients compared to HCs
						mtDNA presence in SF correlated significantly with rheumatoid factor positivity
Collins et al. (129)	2004	RA (54)	17	PCR and SDS–polyacrylamide-gel electrophoresis	SF	PCR-amplifiable mtDNA fragments detected in SF of RA patients but not in HCs
Zhong et al. (156)	2007	RA (54)	44	qPCR on purified cfDNA	Serum and plasma	↑ concentrations of cfDNA in RA patients compared to HC
						↑ serum-to-plasma cfDNA ratio in RA patients compared to HC
						↑ antibody-bound plasma cfDNA in RA patients compared to HC
Chen et al. (5)	2007	SLE (12)	8	PicoGreen assay (fluorescence detection) on purified cfDNA	Serum and Plasma	↑ concentrations of cfDNA in SLE patients compared to HC
						↑ concentrations of cfDNA in the serum compared to plasma
Bartoloni et al. (157)	2011	SLE (44)	66	qPCR on purified cfDNA	Plasma	↑ cfDNA in SLE, RA and SS
		RA (20)				Correlation of cfDNA with disease activity in SS
		SS (48)				
Cepika et al. (158)	2012	SLE (15)	11	qPCR on purified cfDNA	Serum	↑ concentrations of cfDNA in SLE patients compared to HC
						↑ concentrations of cfDNA in SLE patients compared to HC following treatment.
						↓ cfDNA levels in chloroquine treated patients compared to untreated patients
Tug et al. (159)	2014	SLE (59)	59	qPCR on unpurified cfDNA	Plasma	↑ concentrations of cfDNA in SLE patients compared to HC
						No difference in the DNA integrity between SLE and HC
						cfDNA levels fluctuate with disease activity
Zhang et al. (42)	2014	SLE (54)	43	PicoGreen assay (fluorescence detection) on unpurified cfDNA	Plasma	↑ concentrations of cfDNA in SLE patients compared to HC
						↑ concentrations of cfDNA in SLE patients with LN compared to patients without LN
						↑ concentrations of cfDNA in SLE patients with active LN compared to patients with inactive LN
Chan et al. (19)	2014	SLE (24)	11	qPCR on purified plasma DNA	Plasma	↑ aberrant genomic representation, size shortening and hypomethylation of plasma DNA
				Plasma DNA sequencing and methylation analysis		Correlation with SLEDAI and anti-dsDNA antibodies
Hendy et al. (160)	2015	SLE (52)	25	qPCR on purified cfDNA	Serum	↑ concentrations of cfDNA in SLE patients compared to HC
						cfDNA levels fluctuate with treatment
Dunaeva et al. (6)	2015	RA	29	qPCR on purified cfDNA	Serum	↓ cfDNA levels in esRA compared to eRA, RRMS and HC
		eRA (39)				cfDNA levels in eRA, RRMS comparable to HC
		esRA (26)				
		RRMS				
Abdelal et al. (161)	2016	SLE (35)	25	qPCR on purified cfDNA	Plasma	↑ concentrations of cfDNA in SLE patients compared to HC
		RA (30)				Correlation with ESR, anti-dsDNA, C3, C4 and SLEDAI-2000
Rykova et al. (162)	2017	RA (74)	63	qPCR on purified cfDNA	Plasma and cell-surface bound	↑ concentrations of plasma nuclear DNA in RA patients compared to HC.
						No differences in plasma mtDNA levels between RA patients and HC
						↑ concentrations of cell-surface bound mtDNA and ↓ levels of cell-surface bound nuclear DNA in RA compared to HC
Hashimoto et al. (163)	2017	RA on bDMARD (30)	21	qPCR on purified cfDNA	Plasma and SF	↑ concentrations of plasma cfDNA in RA patients compared to HC
		OA (12)				Compared to baseline ↑ concentrations of plasma cfDNA in RA patients with the introduction of biological DMARDs until 8 weeks
						and is associated with improvement in disease activity
						↑ concentrations of SF cfDNA in RA patients compared to OA patients.
Laukova et al. (164)	2018	RA on bDMARD (37)	none	qPCR on purified cfDNA	Plasma	↓ in total cfDNA, nuclear and mt DNA 6 months post-bDMARD treatment and association with clinical and laboratory parameters
Eldosoky. et al. (165)	2018	RA (35)	22	qPCR on purified cfDNA	Plasma	
Xu et al. (166)	2018	Pregnant women with SLE (36)	199	Fluorometric Qubit^®^ dsDNA BR Assay Kit Qubit assay	Plasma	↑ levels of cfDNA in non-pregnant and pregnant women with SLE compared to HC
		Non-pregnant women with SLE (22)	60			↑ levels of cfDNA in patients with active SLE compared to patients with inactive disease
						Correlation of SLEDAI scores with higher cfDNA levels in entire patient cohort, non-pregnant and pregnant patients

*Anti-dsDNA, Anti-double stranded DNA; bDMARD, Biological disease-modifying antirheumatic drugs; C3, Complement factor C3; C4, Complement factor C4; DM, Dermatomyositis; eRA, Early RA; ESR, Erythrocyte sedimentation rate; esRA, Established RA; HC, Healthy control; MtDNA, Mitochondrial DNA; OA, Osteoarthritis; qPCR, Quantitative real-time PCR; RA, Rheumatoid arthritis; RRMS, Relapsing-remitting multiple sclerosis; SF, Synovial fluid; SLE, Systemic lupus erythematosus; and SLEDAI, SLE disease activity index*.

## Early Reports of cfDNA Detection in SLE

In 1948, Mandel and Metais were the first to report on the presence of cfDNA in human plasma (1). However, it was not until the discovery of high levels of cfDNA in SLE patients in 1966 by Tan et al. (2), that the interest in cfDNA as a potential biomarker for autoimmune diseases started. Tan et al. (2) used SLE sera with a precipitating antibody to DNA in a gel diffusion method to detect the presence of DNA in pathological sera. By this method, native (ds) DNA could be detected in sera of some SLE patients (11/95) and in patients with other disease conditions but not in the sera of healthy controls. Gel precipitation has a detection limit of 1 μg/ml; hence it is possible that samples with lower levels of cfDNA, especially of healthy controls could have gone undetected. Later, in 1968 Barnett used complement-fixation, to demonstrate the presence of cfDNA in normal and pathologic human sera and synovial fluids (3). Sera of patients positive for precipitating antibodies to DNA and having complement-fixing antibodies to DNA were used to detect DNA in human samples. Unlike Tan et al. (2), small but detectable amounts of DNA could be measured in normal sera. However, the levels of cfDNA were markedly elevated in pathologic sera and synovial fluid. In 1973, Koffler et al. (4), by hemagglutinin inhibition test, reported that about 50% of SLE sera are positive for the presence of single-stranded DNA (ssDNA) as an antigen. In contrast, ssDNA appeared infrequently in the sera of healthy controls (4% incidence). Almost at the same time, Davis and Davis (147), used a counterimmunoelectrophoresis (CIE) method to detect cfDNA in the plasma of patients with various illnesses including SLE. The number of positive samples in SLE patients (2/47, 4.2%) were not significantly different from healthy controls (1/83, 1.2%).

## The Controversy of Detection Methods and Serum vs. Plasma Utility for cfDNA Measurement

At the time when the clinical importance of cfDNA was being actively pursued, conflicting data on the detection of cfDNA in the circulation of healthy individuals led to controversies on the detection methods and on the significance of serum and plasma cfDNA levels for disease. In addition, a consensus was yet to be reached on the levels of cfDNA in plasma for normal vs. pathological scenarios. Davis and Davis (147), with their method of CIE, were the first to hint that cfDNA in sera, at least in part, could be an artifact of the method, i.e., DNA that is sporadically released into sera during blood clotting. This observation was confirmed by Steinman (149) in an elegant study that employed four different techniques with improved sensitivities and/or specificities to detect cfDNA in normal serum and plasma samples. In that study, plasma cfDNA remained undetectable by all four methods including a highly sensitive CIE method. Further, it was reported that the cfDNA measurements in plasma by ethidium bromide and diphenylamine assays, are mainly due to interfering substances (non-DNase sensitive substances) in plasma rather than true DNA. In contrast, cfDNA could be detected in serum samples by a CIE method. Based on these findings, it was concluded that cfDNA detection in plasma is pathological and levels >50 ng/ml for dsDNA and 100 ng/ml for ssDNA in plasma are abnormal. These findings were later confirmed by Dennin (167), who, by employing CsCl-buoyant density centrifugation found that in healthy adults the concentration of plasma cfDNA ranged from 3 to 11 ng/ml (167). In more recent times, Chen et al. (5), showed that serum samples from lupus patients had higher levels of cfDNA than the corresponding plasma samples by using a fluorochrome PicoGreen assay on purified cfDNA. A higher sera-to-plasma cfDNA ratio suggests that white blood cells from SLE patients are fragile and/or damaged and thus are prone to undergo disruption during coagulation releasing DNA. These studies strengthened the view that cfDNA levels from serum samples should be interpreted with caution especially when employing sensitive detection methods and if possible, should be replaced by carefully collected plasma samples.

## cfDNA and Disease Activity in SLE

The majority of studies reported an association between cfDNA and disease activity in lupus. However, there are a few studies with conflicting data on the link between cfDNA and disease activity (152, 154, 160, 168). Early reports by serial sampling of cfDNA from sera demonstrated that an increased appearance of cfDNA in SLE patients is associated with the exacerbation of disease and interestingly becomes undetectable following the clinical improvement (2, 4, 153). Koffler et al. (4) in a serial study of 18 SLE patients, followed for periods of 6–51 months observed that in certain patients, cfDNA (ssDNA) can reach extreme high levels in the range of 125–250 μg/ml. Similar to Tan et al. (2), ssDNA antigen appearance was associated with episodes of clinical exacerbations and patients with prolonged presence of ssDNA (4–8 months) reported progressive renal disease (4). Evidence for *in vivo* antigen-antibody formation was demonstrated where ssDNA antigen appearance alternated or occurred simultaneously with anti-ssDNA antibodies. Morimoto et al. (153) observed a highly significant correlation between DNA derived from circulating immune complexes and disease activity index in SLE patients. A serial study of two patients demonstrated that the levels of cfDNA remain elevated (52 ng/ml) during the episodes of active disease and glomerulonephritis (100 ng/ml) and return to lower levels during clinical remission (10 ng/ml) and further becoming non-detectable with treatment (<1 ng/ml). Steinman (149), observed that majority of SLE patients (80%) with central nervous system involvement and/or systemic vasculitis have a persistent presence of cfDNA in plasma. Longitudinal studies in four SLE patients also confirmed this association with CNS and/or vasculitis, where only episodes associated with these manifestations are characterized by cfDNA appearance. Raptis et al. (150), showed that cfDNA exists in much higher levels in the plasma of untreated SLE patients with active disease compared to plasma of patients with corticosteroid-induced disease remission and healthy controls. A serial determination of plasma in SLE demonstrated that cfDNA levels are elevated at disease onset and diminished considerably when disease has stabilized accompanied by a concomitant decrease in the serum dsDNA binding activity. Tug et al. (159), though not observing a clear link between SLE disease activity index (SLEDAI) and cfDNA concentrations, found a significant correlation between fluctuations in cfDNA levels and transition from remission to deteriorating status. This study suggested that changes in disease state, in particular deterioration status, could be reflected by fluctuations in cfDNA (159). Zhang et al., (42), investigated the proposition that elevated levels of plasma cfDNA in SLE are related to lupus nephritis (LN). cfDNA concentrations were significantly higher in SLE patients with LN than in patients without LN and further subgrouping analysis revealed that patients with active LN had significantly elevated cfDNA concentrations compared to patients with inactive LN. Studies in SLE patients also reported that cfDNA levels correlate significantly with SLEDAI (53, 166). A recent study by Xu et al. (166), showed that median cfDNA levels are significantly higher in SLE patients with active disease compared to patients with inactive disease.

## cfDNA and Treatment Response in SLE

Considering that inflammation can cause the release of cfDNA and the fact that cfDNA itself can perpetuate ongoing inflammation leading to a vicious feedback loop, a drug treatment that reduces systemic inflammation or specifically antagonizes receptors that recognize DNA, would likely affect cfDNA levels as well. Currently, literature demonstrating the dynamics of cfDNA levels in SLE patients with treatment is sparse, but promising. Hendy et al. (160) observed that SLE patients following specific therapy with cytotoxic drugs show a significant reduction in serum cfDNA levels compared to pre-treatment and this was accompanied by a concomitant reduction in anti-dsDNA levels and anti-nucleosome antibodies. Cepika et al. (158) showed that sera cfDNA levels in SLE patients decrease significantly following treatment with chloroquine, a drug known to block the DNA-sensing TLR9 pro-inflammatory pathway. Although not significant, corticosteroid (CS) treatment also decreased serum cfDNA levels, suggesting that reduction in systemic inflammation decreased cfDNA levels. In contrast, in another study (157), the type of treatment, CS and/or immunosuppressive (IS) did not seem to affect the levels of plasma cfDNA in SLE patients.

## cfDNA and Association With Inflammatory Markers in SLE

cfDNA has been receiving increasing attention as an inflammatory marker with the advent of new mechanistic studies highlighting the role of DNA sensing receptors in SLE pathogenesis (146). Hence, attempts were made to evaluate the relationship of cfDNA with other known markers of inflammation. Overall, although limited by the number of studies, it can be concluded that cfDNA levels in SLE associate well with several markers of inflammation. One study found a significant positive correlation between serum cfDNA levels and a generic marker of inflammation, CRP (149). NETs released from neutrophils and low-density granules (LDGs), could also be the source of cfDNA in SLE. Consistent with this assumption, Zhang et al. (42) found a highly significant positive correlation between levels of plasma cfDNA and the percentage of LDGs and neutrophil levels, suggesting that LDGs and neutrophils, through NET formation, can contribute to cfDNA in SLE patients. However, the authors never confirmed whether cfDNA levels correlated with presence of circulating NETs in these patients. In addition to the abnormal production of NETs, impaired clearance of NETs can also lead to elevated levels of cfDNA in SLE. Further analysis demonstrated a significantly lower DNase I activity in SLE patients compared to healthy controls, although no significant correlation could be observed between DNase I activity and cfDNA levels (42). Studies found contrasting associations for the levels of complement factors and cfDNA in SLE patients. Tug et al. (159) and Abdelal et al. (161) found a positive correlation between the levels of complement factors and plasma cfDNA levels in SLE patients. This observation was surprising given that complement consumption is commonly seen in active SLE patients. However, the authors speculated that this positive relationship between complement levels and cfDNA might be due to the increase in complement levels as a part of an acute phase response that can obscure the complement consumption. In contrast, a study by Hendy et al. (160) found a significant negative correlation between levels of serum cfDNA and C3 in SLE patients.

Based on the central role of anti-nuclear antibodies in SLE, and the possibility that high levels of cfDNA in circulation might initiate and/or perpetuate the production of anti-dsDNA antibodies, a positive correlation between cfDNA levels and anti-dsDNA antibodies is expected. However, in contrast, studies reported either a lack of or negative correlation between cfDNA and anti-dsDNA antibody levels. McCoubrey et al. (154) and Hendy et al. (160) observed an inverse correlation between levels of plasma DNA in SLE patients and titers of antibody for DNA. In another study (157), cfDNA in plasma did not correlate with titers of anti-dsDNA antibodies. This situation can likely arise due to the accelerated tissue deposition of immune complexes during active disease. A serial sampling might provide a better picture of the association between levels of cfDNA and anti-dsDNA antibodies.

## Qualitative Features of Plasma cfDNA in SLE

The SLE genome exhibits distinct qualitative features such as, a higher frequency of CpG dinucleotides (169), increased hypomethylation (170, 171), and increased oxidation (172). Interestingly, all these qualitative features promote the immune stimulatory properties of DNA to be recognized by DNA sensing receptors and subsequently induction of pro-inflammatory responses. Hence, it appears that cfDNA released from SLE patients is inherently proinflammatory and, therefore analyzing the qualitative changes of cfDNA might provide more in-depth understanding on the association of cfDNA with SLE pathogenesis and eventually it's utility as a biomarker for SLE. However, in SLE literature only one study has been reported investigating the qualitative changes of plasma cfDNA (19), warranting the need for more studies in this area of cfDNA research. Chan et al. (19) by a parallel genomic and methylomic sequencing observed various abnormalities in plasma DNA from SLE patients including aberrant measured genomic representations (MGRs), size shortening, fragments of <115 bp in size, and hypomethylation. Very interestingly all these plasma DNA abnormalities, as discussed below, were seemed to be modulated by anti-dsDNA antibodies, pathological circulating markers of SLE. It was observed that the frequency of aberrant MGRs correlated with the levels of serum anti-dsDNA antibodies. Subsequent experiments demonstrated that aberrant MGRs DNAs had increased binding affinity to anti-dsDNA antibodies. Thus, given the ability of IgG to protect DNA from subsequent degradation, DNA molecules with increased dsDNA antibody binding, such as aberrant MGR DNA, may have increased representation in cfDNA analyses due to their reduced clearance. The percentage of plasma DNA shortening in SLE correlated positively with SLEDAI and the anti-dsDNA antibody, suggesting that there is either an increased release or decreased clearance of short fragments in SLE. This observation aligns well with the evidence of increased apoptosis as well as defective clearance in SLE patients (17, 173). Further, IgG-bound DNA was enriched in short fragments (<115 bp), strengthening the proposition that IgG antibody has a preferential binding to short DNA fragments, that in turn protect them from degradation. Plasma DNA molecules from active SLE patients were more hypomethylated compared to inactive SLE and healthy controls. In addition, the degree of hypomethylation correlated with SLEDAI and anti-dsDNA antibody levels. Based on size distribution profiles and methylation density, it was suggested that plasma DNA in SLE patients exhibit size shortening with hypomethylation and are protected from degradation by antibody-binding.

## Summary of cfDNA Research in SLE

Overall, SLE patients show elevated levels of cfDNA that fluctuate concomitantly with disease activity, inflammatory markers and to some extent with therapeutic interventions. The association of cfDNA with existing SLE diagnostic marker, anti-dsDNA antibodies, is unclear with conflicting data, suggesting that the dynamics of cfDNA in SLE is independent of anti-dsDNA antibodies. cfDNA also seems to reflect the genomic modifications characteristic of SLE disease. However, there are many factors that needs to be addressed in order to establish cfDNA as a biomarker for SLE from a clinical standpoint. cfDNA quantification as a diagnostic marker for SLE is promising but lacks clinical specificity since it is detected in other diseases albeit at lower levels. Findings from epigenomic research on cfDNA are critical in establishing the clinical biomarker specificity of cfDNA for SLE. Mechanistic studies of SLE pathogenesis have demonstrated an enhanced and preferential release of proinflammatory oxidized mitochondrial DNA into circulation by SLE neutrophils (38). Interestingly, none of the SLE-cfDNA studies have analyzed mitochondrial cfDNA in SLE patients. Given the preferential release of mitochondrial DNA in SLE, the quantification and characterization of mitochondrial DNA, and its relative abundance to nuclear DNA, will likely provide disease specific information on cfDNA in SLE. Apart from being a diagnostic marker, cfDNA might also function as a broad disease management marker for SLE, such as a marker of prognosis for remission, flare and/or treatment response. However, it requires a rigorous evaluation of cfDNA in longitudinal studies with large cohorts of patients and careful comparison with existing inflammatory and clinical makers of disease.

### Rheumatoid Arthritis

RA is, similar to SLE, an autoimmune rheumatic disease primarily affecting joints, with severe and disabling erosion (174). Though not as frequent as in SLE, also RA patients have been reported to develop anti-DNA antibodies (175). This has significance given the ability of DNA immune complexes to engage both antigen receptor and TLR9 simultaneously, inducing B cell proliferation and antibody secretion as seen in rheumatoid factor (RF) expressing B cells (176). Further, mtDNA and/or oxidized nucleic acid material was able to induce arthritis in mice (129). In addition to the above-mentioned evidence of DNA in RA, the potential release of cfDNA in general during inflammation, have led to many studies exploring the potential of cfDNA as a biomarker of diagnosis, disease activity and progression, and/or treatment response in RA. Major findings of cfDNA studies in RA are summarized and listed in Table 1.

## cfDNA and RA

Similar to SLE, the majority of studies reported elevated levels of circulating cfDNA in RA patients compared to controls (3, 4, 155, 156, 161–165). Cell-free nuclear and mtDNA content of synovial fluid in RA patients was reported to be many folds higher than corresponding plasma levels and was exclusive to patients, suggesting that cfDNA release in RA patients is mainly localized to the joints and is pathologically relevant (151, 155, 163). Previous investigations of Rykova et al. (162) in cancer, demonstrated that cfDNA levels in whole blood are a result of continuous exchange between free DNA and cell-surface bound (csb) DNA, and that free DNA binds to cells via direct binding to cell surface proteins and through plasma proteins. In their study on RA patients, Rykova et al. (162) found contrasting dynamics of free- and csb-DNA forms for nuclear and mtDNA compared to controls. While plasma mtDNA levels were not significantly different between RA and healthy controls, csb-mtDNA levels were significantly elevated in RA patients. In contrast, plasma nuclear DNA levels were significantly elevated in RA patients, with a significant decrease in the csb-nuclear DNA levels. The finding on csb-mtDNA is interesting given its role in inflammation, and the decrease in the levels of csb-nuclear DNA could be due to disease-induced changes in the composition of circulating nuclear DNA-protein complexes in RA patients, that might have influenced the nuclear DNA binding to cells (162). In contrast to prior studies, Dunaeva et al. (6) reported that serum cfDNA levels were comparable between patients with early RA (eRA) and healthy controls. Furthermore, patients with established disease (esRA) have significantly lower levels of serum cfDNA compared to patients with eRA and healthy controls. Levels of cfDNA in serum did not correlate with serum DNase activities, suggesting that lower cfDNA levels in esRA is not due to elevated DNase activities. Lower levels of cfDNA in esRA patients could be due to treatment with disease-modifying drugs that are known to reduce systemic inflammation and subsequent cfDNA release. Nevertheless, this study suggested that serum cfDNA can be used as a disease progression marker in RA patients (177).

## High Serum-to-Plasma cfDNA Ratio in RA

Consistent with the prior observations in SLE, RA patients also exhibited a higher serum-to-plasma cfDNA ratio compared to healthy controls (156), implying that leukocytes in pathological conditions, in general, have an altered susceptibility to undergo cellular death and cfDNA release upon clotting. In an independent study authors evaluated if NETs are the source of higher cfDNA levels in sera of RA patients, based on their *in vitro* findings that neutrophils from RA patients were prone to undergo excessive NETosis. Accordingly, analysis of sera and corresponding plasma samples revealed that, sera from RA patients and not the plasma, have increased concentrations of cfDNA and NET-derived components compared to healthy controls. This observation suggested that coagulation during serum preparation triggers an extensive NETosis in RA neutrophils releasing NETs that in turn contribute to the elevated levels of cfDNA in serum (41).

## cfDNA and Serological Parameters in RA

Evidence for the association of cfDNA with serological parameters of RA, including RF and anti-citrullinated protein antibodies (ACPA), is sparse, with conflicting data. Leon et al. demonstrated that higher levels of serum cfDNA in RA patients correlate with seronegativity (148). Consistent with the susceptibility of mtDNA to undergo oxidation, Hajizadeh et al. (155) found that patients that were positive for mtDNA in SF also had high levels of 8-OHdG, a marker of oxidative status. In contrast to Leon et al. (148) both cell-free mtDNA positivity and levels of 8-OHdG correlated significantly with rheumatoid factor positivity. Rykova et al. (162) found a negative correlation between ACPA and plasma mtDNA levels in RA patients. These data suggest that cfDNA and ACPA/RF could be independent circulating makers of RA development and their combination might result in an improved diagnostic tool for RA.

## cfDNA and Disease Activity in RA

In a majority of studies, cfDNA levels in RA patients were reported to be associated with disease activity and markers of inflammation (148, 151, 156, 161). DNA levels measured in paired samples of serum and synovial fluid (SF) from patients with arthritides [seropositive RA, seronegative RA variants including psoriatic arthropathy, ankylosing spondylitis and juvenile RA, gout, pseudogout, osteoarthritis (OA), and post-traumatic arthritis (TRA)], demonstrated that the RA patients, as well as patients with gout and pseudogout had the highest levels of cfDNA in SF and serum. Very low levels of cfDNA were seen in patients with OA and TRA. A serial determination of cfDNA revealed a temporal correlation between the elevated levels of DNA in serum and SF and parameters of disease activity and inflammation in some RA patients (151). Abdelal et al. (161) showed that elevated levels of plasma cfDNA in RA patients correlated significantly with erythrocyte sedimentation rate (ESR), CRP and Disease Activity Score-28 (DAS28, suggesting that plasma cfDNA can be a potential marker of disease activity in RA patients. In contrast, Hajizadeh et al. (155) did not find any obvious association between cell-free mtDNA in SF and markers of disease activity and severity, including extra-articular manifestations, erosion, leukocyte counts, CRP levels, or disease duration. Similar to SLE, the majority of plasma cfDNA in RA patients was found to be associated with antibody (156), suggesting the role of DNA-anti-dsDNA immune complexes in RA pathogenesis. More recently, Eldosky et al. (165) found that in RA patients, cfDNA levels are significantly higher in active disease group compared to control group, while the levels are comparable between remission and control groups. ROC curve analysis revealed a sensitivity and specificity of 86 and 84%, to differentiate active and remission states of RA. Correlation analysis in all RA patients showed that cfDNA levels correlate significantly with DAS28-ESR, a marker of disease activity. In addition, cfDNA showed an inverse correlation with absolute lymphocyte count, suggesting a possible role of enhanced lymphocyte death in RA patients (178) including NETosis, in the formation of cfDNA. Overall, the study suggested that cfDNA can be potential marker of disease activity progression in RA (165).

## cfDNA and Treatment Response in RA

In RA patients, changes in cfDNA levels following the treatment with biological disease-modifying anti-rheumatic drugs (bDMARDs), were associated with the improvement in disease activity. Hashimoto et al. (163) reported that in a subset of RA patients, plasma cfDNA can be a predictor of early therapeutic response of bDMARDs. Specifically, a cfDNA elevation at 8 weeks can predict the therapeutic response of biological bDMARDs from 12 to 24 weeks. At baseline, plasma cfDNA levels in RA patients were significantly higher than the healthy controls. After the introduction of DMARDs, the cfDNA increase at 8 weeks was associated with a concomitant improvement in average SDAI score and disease activity. A study by Laukova et al. (164), further investigated the effect of bDMARDs on plasma cfDNA with regards to it subcellular origin, nuclear, and mitochondrial, respectively. This study, in contrast to Hashimoto et al. (163) demonstrated that plasma cfDNA decreases following dDMARDs therapy. Plasma samples were analyzed for cfDNA content from RA patients at baseline as well as 3- and 6-months post-bDMARD treatment. There was a clear improvement in clinical (DAS28, swollen and tender joints) and laboratory parameters (ESR, CRP) in all patients by 3 months following bDMARD therapy. Although the levels of total cfDNA, nuclear, and mitochondrial cfDNA started to decrease by 3 months, significant differences from baseline were observed only 6 months post-treatment, where the concentrations decreased by half. These observations were in contrast to Hashimoto et al. (163), where an increase in plasma cfDNA was observed until 8 weeks post-bDMARD treatment. However, in the absence of additional time points between baseline and 3 months, it is not possible to rule out the dynamics of cfDNA levels in the Laukova et al (164), study as well. Laukova et al. (164) further observed that in RA patients, lower concentration of total cfDNA, correlated positively with DAS28, ESR and CRP. No differences were found between good responders and moderate responders in the levels of total cfDNA, nuclear and mitochondrial cfDNA pre- and post-bDMARDs treatment. In good responders, the concentration of total cfDNA and nuclear cfDNA decreased significantly 6 months from the baseline. Since the decrease in cfDNA levels following bDMARDs therapy is much slower in comparison to other routinely measured laboratory and clinical parameters, the decrease in cfDNA was interpreted as a consequence of reduced inflammation due to treatment. On similar lines, Zhong et al. (156), observed that plasma cfDNA levels were markedly changed (either increase or decrease) in 7 out of 10 patients, 1 h after infusion of infliximab. In another study, a group of RA patients treated with rituximab showed a tendency of lower cell-surface bound mtDNA levels than a group treated with methotrexate and etoricoxib, although the difference did not reach statistical significance (156).

## Summary of cfDNA Research in RA

To summarize, RA patients in general have elevated levels of circulating cfDNA and in SF, cfDNA is found at concentrations many times higher than in circulation, suggesting the role of localized inflammation in the release of cfDNA. Further, the detection of cfDNA exclusively in the joints (SF) of RA, strengthens the arthritic potential of cfDNA. Association of cfDNA with seropositivity is unclear with conflicting results and, the quantitative changes in cfDNA seem to reflect the disease progression and treatment response in RA. Data suggest that dynamics of cfDNA in RA patients is independent of existing diagnostic markers, ACPA and RF, and cfDNA in combination with ACPA/RF might form an improved diagnostic tool. Although, the role of mtDNA in arthritis is demonstrated (129), only two studies have quantified the levels of mtDNA in the circulation of RA patients, highlighting the need for more research to explore the role of circulating mtDNA as a biomarker for RA. While, the studies demonstrate the biomarker potential of cfDNA in RA, longitudinal studies with large cohorts of patients are needed to capture the dynamics of cfDNA in RA with disease progression and drug effects.

## Comparison of cfDNA Observations Between SLE and RA

Considering the many technical and sampling variations in different studies, direct comparisons of cfDNA levels between SLE and RA patients can only be made from studies where both patient cohorts were analyzed simultaneously. In general, SLE patients have higher concentrations of sera cfDNA (ssDNA) along with higher positivity for anti-ssDNA antibodies as compared to patients with RA (4). The association of cfDNA levels with serological parameters in both diseases, e.g., anti-dsDNA in SLE (154, 160) and ACPA and RF in RA (148, 155, 162), suggest that cfDNA may reflect common processes involved in both diseases, including inflammation and cell death. RA patients exhibit increased levels of mtDNA in plasma and SF (155, 162). Given recent studies implicating mitochondrial extrusion in the SLE pathogenesis (38, 88), we expect upcoming studies to demonstrate increased mtDNA levels also in SLE patients. cfDNA from both RA and SLE exhibit increased oxidation (125, 155), possibly a consequence of inflammation. Contrasting dynamics of cfDNA was observed with treatment for SLE and RA with cfDNA levels decreasing in SLE patients upon treatment (158, 160). In RA, however, cfDNA levels initially increase, where after they decrease at later time-points (163, 164). Finally, cfDNA levels associate with markers of disease activity and inflammation in both RA and SLE (2, 4, 42, 148–151, 153, 156–158, 161).

## Recent Advances in Analysis of cfDNA in Other Medical Fields

cfDNA is not only used for chronic inflammatory rheumatic diseases, but also in several other conditions, including non-invasive prenatal testing to detect fetal aneuploidies (179), cancers (180), as an early marker of allograft rejection and graft damage (181) and for tracking microbial infection, including the detection of oncogenic viral DNA (182). Methodological advancements in the analysis of cfDNA are comprehensively addressed in recent reviews (183, 184). In brief, emerging targeted approaches to screen for specific mutations of cfDNA include; digital droplet PCR (ddPCR), a highly sensitive method that allows the identification of rare targets based on the partitioning of samples into water-into-oil droplets; BEAMing (beads, emulsification, amplification, and magnetics) a combination of emulsion PCR and flow cytometry to achieve higher sensitivity; next generation sequencing (NGS), a powerful technique that allows the screening of both targeted and untargeted mutations; and methylated CpG tandem amplification and sequencing (MCTA-Seq) to identify genome-wide hypermethylated CpG regions. Other cutting-edge technologies that allow personalized therapies include targeted plasma re-sequencing (TAm-Seq) and personalized analysis of rearranged ends (PARE) which is based on the identification of disease-specific somatic rearrangements. Fluorescence correlation spectroscopy (FCS), a method based on the fluctuations of fluorescence due to the Brownian movement of fluorescence molecules, allows a rapid and sensitive detection of single molecules, including the size determination of DNA (185). FCS can effectively complement existing methods of cfDNA detection in autoimmune diseases.

## Closing Remarks and Future Directions

The relevance of DNA to the disease pathology of lupus is undisputed and, there is an increasing attention in RA as well. In contrast to non-specific markers of inflammation, cfDNA can be pathologically relevant to autoimmune rheumatic diseases given the role of DNA-sensing receptors in inflammation and autoimmunity. cfDNA allows a rapid, easy, non-invasive and repetitive method of sampling. A combination of these biological features and technical feasibility of sampling, position cfDNA as a potential biomarker of enormous utility for autoimmune rheumatic diseases. However, there are many issues that needs to be addressed toward this goal. It should be acknowledged that the underlying heterogeneity of autoimmune disease by itself, can contribute to a considerable amount of variation in the levels of cfDNA, and hence adequate measures must be taken to minimize the variations at the level of cfDNA sampling. Notably, there is a lack of uniformity on the type of sample (plasma/serum/synovial fluid), methods of sample collection/processing, free or cell-surface bound DNA, cfDNA extraction and cfDNA quantification, and also in the presentation and interpretation of quantitative cfDNA findings. Additional, complexity is brought by the advent of qualitative research of cfDNA, which needs to be standardized as well. Given this lack of homogeneity, it is not surprising that consensus is yet to be reached on cfDNA levels in healthy individuals. Further, the majority of studies have been cross-sectional, and were limited by sample sizes. However, in order to fully understand the biomarker potential of cfDNA in autoimmune rheumatic diseases, a systematic scientific framework with collaborative efforts is needed to conduct large, multicenter trials with prospective analyses.

## Author Contributions

BD and CL conceptualized the manuscript idea. BD conducted the literature search and drafted the manuscript. CL provided critical revisions. Both BD and CL edited the manuscript.

### Conflict of Interest Statement

The authors declare that the research was conducted in the absence of any commercial or financial relationships that could be construed as a potential conflict of interest.
